# Community Preventive Health Education Intervention for Pediatric Iron-Deficiency Anemia in Rural Southeast Nigeria

**DOI:** 10.5334/aogh.3625

**Published:** 2022-11-21

**Authors:** Adaure Nwaba, Michelle Su, Victoria Rajamanickam, Kelechi Mezu-Nnabue, Udo Ubani, E. Uchenna Ikonne, Olachi Mezu-Ndubuisi

**Affiliations:** 1 University of Michigan, Ann Arbor, MI, US; 2 Department of Pediatrics, University of Wisconsin, Madison, WI, US; 3 Department of Biostatistics and Medical Informatics, University of Wisconsin, Madison, WI, US; 4 Mezu International Foundation, Pikesville, MD, US; 5 Department of Optometry, Abia State University, Uturu, Nigeria; 6 Department of Pediatrics, Department of Ophthalmology, University of Rochester, Rochester, NY, US

**Keywords:** anemia, iron deficiency, pediatrics, community education, self-efficacy

## Abstract

**Objectives:**

Iron-deficiency anemia (IDA) is a global cause of morbidity in children under five, particularly in sub-Saharan Africa. In southeast Nigeria, poor dietary intake and caregiver knowledge about childhood anemia are observed; however, there is no consensus on how to best prevent it. This study seeks to test the effectiveness of caregiver education on improving anemia knowledge and dietary prevention strategies and promoting sustainable lifestyle changes to reduce the prevalence of childhood IDA.

**Study Design:**

A questionnaire was administered to the primary caregivers of 41 patients under age five with anemia in southeast Nigeria regarding socioeconomic status (SES), diet diversity, and risk factors for anemia. Caregivers were administered a preeducation questionnaire, poster education on anemia and iron-rich foods, and a posteducation questionnaire. All patients underwent a medical exam to confirm a diagnosis of anemia or anemia-related conditions.

**Results:**

Ninety-five percent of patients had moderate diet diversity, but there was no correlation between diet diversity and SES. Barriers to healthier diets were associated with SES. Preeducation scores were not associated with caregivers’ education levels; however, posteducation scores were significantly higher in university-educated than technical-trained caregivers. Caregiver-reported self-efficacy increased after the education program.

**Conclusion:**

Caregivers’ SES was associated with financial and knowledge barriers to a healthier diet but not diet diversity, suggesting that nutritional education could benefit all SES groups. Overall, the education program increased caregivers’ anemia knowledge across educational levels. A community-based health education program could improve caregivers’ anemia knowledge and self-efficacy in applying this information and potentially reduce this area’s pediatric IDA.

## Introduction

Iron-deficiency anemia (IDA) is a global health crisis and a major cause of morbidity and mortality in children under five years of age [1,2]. According to data from the World Health Organization (WHO), the prevalence of anemia in children under five has remained relatively stable in Nigeria, at around 70%, since 2015, with iron deficiency being the most common cause, both nationally and globally [3]. The prevalence of pediatric IDA in Nigeria is significantly higher than global estimates of 42% for the same age range [4].

Children with IDA, especially children under five, are at increased risk of cognitive deficiencies, growth retardation, learning disabilities, malnutrition, and related conditions such as malaria and hookworm infection [1,5,6,7]. Efforts to mitigate these effects have included guidelines supporting recommendations for either daily or weekly iron supplementation, and in recent years, national food fortification programs [8,9,10,11,12]. However, there has been no consensus on the most effective mode of mitigating this micronutrient deficiency in low- and middle-income countries (LMICs), being limited by a sufficient delivery method, sustainability, and loss to follow-up. Interventions to increase the use of iron-fortified foods [9,13,14] and behavioral-based approaches utilizing community resources [15,16] have been shown to be effective in reducing IDA. However, they require a consistent mode of providing information and access to the populace, which can be challenging in resource-limited areas. Further, sustained behavioral change has been associated with ensuring high rates of self-efficacy in the target population [16,17,18]. Thus, they are adequately supported to be independently capable of implementing the suggested behavioral changes regularly.

The Mezu International Foundation (MIF), a nonprofit dedicated to humanitarian services, runs an outpatient clinic in a rural community in Imo State, Nigeria. It has dedicated resources and research, in collaboration with academic institutions in the United States and Nigeria, to address the most prevalent health concerns of the community it serves. In a 2015 research study, the prevalence of anemia in children aged two to five years in this community was noted to be 69% [19] with a follow-up study in 2017 showing increased prevalence of 80% in children aged one to five years [20], ranges that are comparable to the most recent national prevalence reported by WHO in 2019 [3]. These studies also found that children with lower iron dietary intake were more anemic than older children [20]. In addition, although most families were of middle socioeconomic status (SES), primary caregivers overall had poor knowledge of anemia, its contributory factors, and the iron status of their children, with 94% of them unaware of the causes of anemia [20].

The lack of awareness of both anemia and its causes, and the impact of diet on causing anemia in rural communities of southeast Nigeria, indicated that a family-centered, behavioral-based nutrition intervention program may help alleviate the burden of illness. Nigeria has implemented initiatives at the national level to improve the fortification of foods with iron, such as wheat flour and other grains, in an effort to combat the high rates of micronutrient malnutrition [8,21]. A longitudinal education-based intervention can increase knowledge of iron fortification of foods in this community, decrease the prevalence of pediatric IDA, and increase health literacy on anemia.

Our study aims to assess the effectiveness of an education-based community intervention to increase access to knowledge on IDA and iron-fortified staple foods to caregivers. This study would serve to increase caregivers’ knowledge of anemia and self-efficacy or capability in implementing the information provided in the intervention.

## Methods

The study adhered to the guidelines of the Declaration of Helsinki and was approved by the Institutional Review Board of Abia State University’s Department of Optometry, Uturu, Abia State, Nigeria. This study was implemented during one of the annual medical missions hosted by the MIF at its outpatient health facility in Imo State, Nigeria, wherein patients are offered free health care services and resources. Informed consent was obtained by caregivers for all patients prior to participating in the study. The research design utilized was a single-subject research design aimed at assessing the effect of our educational intervention on caregivers’ knowledge and self-efficacy related to preventing and treating pediatric IDA. This was best suited to assess the interval short-term changes as a result of our intervention and to ensure that differences, such as educational background, could be properly accounted for in our analysis.

### Design of questionnaire

A demographic questionnaire was designed to evaluate patients’ medical history and diet diversity and their caregivers’ SES and education level. The SES and diet diversity scores had been developed and validated in a previous study in the same community [19]. The diet diversity score was determined from a questionnaire designed to determine the ratio of food groups and micronutrients in a family’s diet in the previous 24 hours and last week.

The pre-education and post-education surveys included question sets to yield a total anemia knowledge score and a capability score (Appendix 1). A total anemia knowledge score was determined from the first half of the Appendix 1 question set, questions 1–5. It was designed to determine caregivers’ understanding of risk factors, causes, and treatments of anemia. The anemia knowledge score was defined as the total of the five items. A capability score was determined from the latter half of the question set, questions 6–8. It was designed to assess caregivers’ self-reported ability in preventing or treating pediatric IDA and changing their households’ diets. Each item ranged from very capable/independent (1) to will not make a difference (5). The capability score was defined as the total of the three items. A lower capability score indicates that the caregiver has a higher self-efficacy or confidence in their own abilities for the identified tasks. Finally, caregivers were surveyed on which factors would affect how they would add iron-rich foods to their families’ diets after the health education program, question 9. These results are reported in Table 1.

**Table 1:** Caregivers’ response to “What factors will determine which iron-rich foods you will add to your child’s diet?” pre- and post-education seminar.


RESPONSE OPTION	PRESEMINAR	POSTSEMINAR

**Availability**	14	12

**Price**	13	10

**Already a part of diet/cooked at home**	8	10

**Easy to cook/add to the diet**	1	3

**Easy for the child to eat**	5	6


A health education seminar was designed to educate caregivers on the causes, risk factors, and treatment options for anemia, including diet-specific changes with food from their region. Caregivers attended a poster presentation (Appendix 2) and were provided a brochure to take home (Appendix 3). The pre-education survey was provided to caregivers before the program, and the post-education survey was provided after the program (Appendix 1).

### Subject selection

Forty-one subjects between 5 and 60 months of age were selected to participate in the study. Patients were identified during the initial triage of medical mission participants. All patients had basic demographic information and vitals taken. For those patients between 5 and 60 months of age, their hemoglobin level was additionally taken after informed consent was received from their caregivers. Inclusion criteria was a diagnosis of anemia, as determined by a hemoglobin level <11 per WHO standards using a point-of-care testing tool. The pediatric subjects then underwent a full medical examination by a licensed medical physician for the diagnosis of comorbid medical conditions, and their caregivers completed the study questionnaires and educational program.

### Health education program delivery

The data was collected in a newly constructed outpatient health center being used as field clinic by the nonprofit organization. The data was collected over three days of the free medical outreach. The first author administered the surveys and delivered the education seminar to all caregiver groups. The presenter was aided by an Igbo language interpreter, as the indigenous language is Igbo, although participants reported speaking and reading English. The interpreter was available to repeat questions and instructions in the native Igbo language, even if participants could speak English, to ensure the context was understood. This also helped endure validity of the study. Clinical staff and students of Abia State University’s Department of Optometry assisted with data collection for this study. Specifically, clinical staff and optometry students aided in collecting demographic information from caregivers, hemoglobin testing of pediatric patients, and ensuring full completion of each survey by caregivers. Students were supervised by the first author and senior authors at all times.

Each health education seminar was delivered when there were about five to ten caregivers present who had completed their and their children’s health appointments during the medical mission. Caregivers of research participants completed the pre-education survey before completing the anemia knowledge education seminar. The participants then engaged in a 20-minute health education seminar in small groups of about five to ten individuals presented by the lead researcher with the same Igbo interpreter and poster each time. After the seminar, the same questionnaire as a post-education survey was readministered to compare pre- and post-education survey results. Survey completion took participants about 5–10 minutes each time (Appendix 1). The health education seminar utilized a large poster board (Appendix 2) that included visual aids, illustrations, and pertinent information on pediatric IDA, with a focus on the main causes and risk factors in this region, effects of anemia, local iron-rich foods, and sustainable ways to make nutritionally optimized meals. Additionally, a brochure that included a summary of this information was provided to caregivers to take home (Appendix 3). The surveys, brochures, poster board, and health education seminar were all developed and administered in English. An Igbo interpreter was present for the duration to translate the seminar into Igbo and answer any clarifying questions of the survey.

Reliability was ensured in this process by having the first author present the health education seminar each time and by ensuring that all participants fully completed the survey responses. The responses to the questionnaire were also checked for validity by repeating the questions and verifying the responses to ensure the questions and responses were clearly understood by participants. Consent was obtained verbally prior to interviewing each participant. The principles under the Declaration of Helsinki were adhered to. The IRB approval number was ABSU/REC/0U/2019/009.

### Data analysis

Data was analyzed using SAS software version 9.4 (SAS Institute, Cary, NC). The paired T-test was used to compare the means of pre- and post-test scores. Correlation, T-test, and ANOVA tests were performed to determine the associations between variables and analyze differences between groups with an alpha <0.05 considered significant. All data are presented as mean±SD unless specified.

## Results

### Demographics

We surveyed the caregivers of 41 children under the age of 5 about the demographic profile of each child and the caregiver’s education and financial background. Our subjects were largely female (63% female, 37% male) with a mean age of 35.2±19.3 months. There was no significant association between the sex of the child and their mean hemoglobin levels (P = 0.22) or number of anemia-related medical diagnoses identified during the medical mission (P = 0.54). Anemia-related medical diagnoses included worm infection, gastroenteritis, and malaria. The child’s age was also not associated with hemoglobin levels (P = 0.21), the total number of anemia-related medical diagnoses (P = 0.05), or outward anemia symptoms (P = 0.74).

### Caregivers had poor knowledge of their child’s diagnosis of anemia

The majority of children (56%) had at least two anemia-related conditions diagnosed during the medical mission that included diarrhea, malnutrition, worms, or malaria. Only 12% of parents (n = 16) were previously aware that their child was anemic. There was no significant association between the last medical visit the caregiver took the child to and their awareness that their child had anemia (P = 0.39). Of the 41 children surveyed, only 31% displayed outward anemia symptoms of tiring easily and/or eating items of non-nutritional value, such as nzu, gravel, or ice. There was no association noted between sex of the child and the number of outward anemia symptoms they were exhibiting (P = 0.10). Neither SES nor the caregiver’s highest level of education received was associated with the caregiver’s knowledge of anemia-related conditions of their child. This association was not statistically significant for a university education compared to secondary education (P = 0.11) or for those with a postsecondary education compared to university (P = 0.38). There was also no association between the caregiver’s knowledge of their child having anemia and their pre-education survey knowledge of anemia (P = 1.68) or their perceived capabilities in being able to prevent IDA (P = 1).

### Diet diversity was not correlated with socioeconomic status

**Figure 1:**
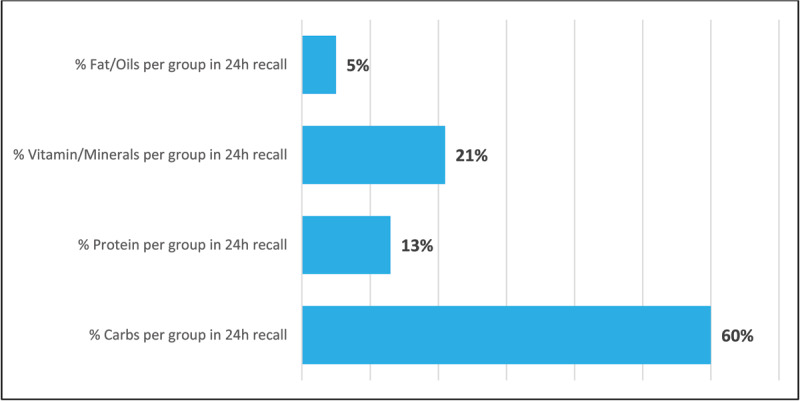
Mean distribution of food group items in family’s diet in last 24 hours.

The amount of diversity in families’ diets was not correlated with SES (P = 0.32), and most caregivers described a moderate diet diversity score (95%, Table 2). The diet diversity scores were also not correlated with the children’s hemoglobin levels (P = 0.21, Table 1) or caregivers’ supplementation of breastfeeding with food (P = 0.68). The distribution of nutrients in their diet for the past 24 hours was as follows: 60% carbohydrates, 13% protein, 21% vitamin/minerals, and 5% fats/oils (Figure 1).

**Table 2:** Diet diversity and hemoglobin levels (mg/dl).*


DIET DIVERSITY	TOTAL NUMBER (N)	HGB LEVEL	SD

**Moderate**	39	9.89	1.44

**Poor**	2	9.25	2.05


Hgb denotes hemoglobin, * denotes means, n denotes number, SD denotes standard deviation.

There was no association between diet diversity score ranking and each caregiver’s perceived barriers to eating a healthier diet (P = 0.33). The predominant barriers reported to eating a healthier diet were as listed (Table 3): they don’t want to make a change (41%), healthier options are expensive (39%), they do not have enough information on healthy diets/do not know what to buy (15%), and they have no access to fresh produce (5%).

**Table 3:** Caregiver-reported greatest barrier to eating a healthier diet compared to post-education seminar caregiver-reported factors that influence how caregivers will add iron-rich foods to their family’s diet.*


CAREGIVER-REPORTED GREATEST BARRIER TO EATING A HEALTHIER DIET	CAREGIVER-REPORTED FACTORS FOR ADDING IRON-RICH FOODS	AVAILABILITY	PRICE	ALREADY A PART OF DIET/COOKED AT HOME	EASY TO COOK/ADD TO THE DIET	EASY FOR THE CHILD TO EAT	TOTAL

**Don’t want to make a change**		4	2	8	2	1	**17**

**Healthier options too expensive**		5	8	2	0	1	**16**

**Not enough information on healthy diets/don’t know what to buy**		1	0	0	1	4	**6**

**No access to fresh produce**		2	0	0	0	0	**2**

**Total**		**12**	**10**	**10**	**3**	**6**	**41**


* Denotes the number of caregivers who selected the indicated barrier/factor.

**Figure 2:**
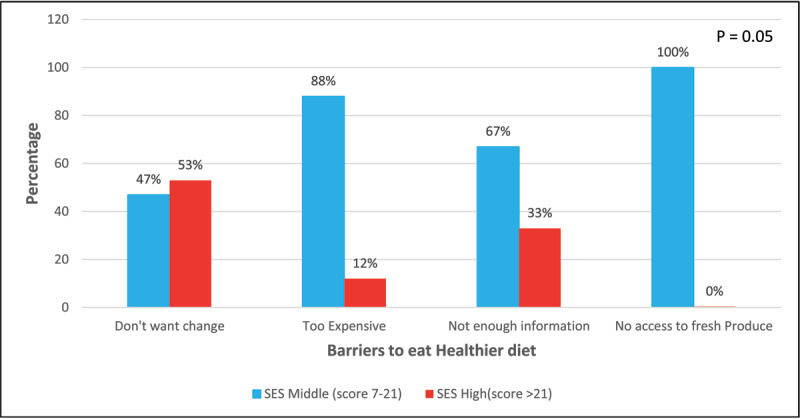
Association between SES and barriers to eating a healthier diet.

SES also did not correlate with what caregivers perceived as their main barrier to eating a healthier diet (P = 0.05) (Figure 2), but it was associated with the driving factor determining how caregivers would add iron-rich foods to their child’s diet both before taking the educational seminar (P = 0.004) and after (P = 0.005). Caregivers were surveyed on which of the listed factors most affected how they would add iron-rich foods to their family’s diet: availability, price, already a part of their diet/cooked at home, easy to cook/add to the diet, and easy for the child to eat (Table 1). Caregivers selected “availability” as the largest driving factor in how they would augment their family’s diet with iron-rich foods both before (n = 12, 29%) and after the seminar (n = 14, 34%) (Table 1). There was also a significant association between their reported barriers to eating a healthier diet (i.e., don’t want to make a change) and the main driving factor for how they would add iron-rich foods to their child’s diet following the educational seminar (P = 0.001).

### Socioeconomic status correlated with local health care utilization

For our cohort, the majority of subjects were of middle SES, with 68% ranked as middle SES and 32% as high SES. SES was associated with how recent the last visit to the doctor was (P = 0.03) but not where families obtained medications (P = 0.13). The possible locations for obtaining medications were from the pharmacy/chemist, either with (73%) or without a prescription (27%); from a friend or relative; or other.

### Post-education survey scores increased after the education seminar

The anemia knowledge portion of the survey consisted of five questions. Caregivers completed the same survey assessing anemia knowledge and caregiver capability before and after the educational seminar (Appendix 1). The mean pre-education score for caregivers was 35±15% and for post-education was 60±22%. The average percent increase in score after the seminar was 25±24%.

For the question, “What causes anemia?” (Table 4), the most selected response was “poor diet” both before the educational seminar (n = 15, 37%) and after the educational seminar (n = 19, 46%). Additionally, the majority of caregivers (n = 40, 98%) indicated they would return for future seminars if made available.

**Table 4:** Caregivers’ response to “What causes anemia?” pre- and post-education seminar.


RESPONSE OPTION	PRESEMINAR	POSTSEMINAR

**Poor diet**	15	19

**Lack of iron in blood**	9	11

**Worms**	0	4

**None of the above**	9	0

**All of the above**	1	7

Don’t know*	7	0


* Caregivers wrote-in “don’t know” on the pretest survey for this response and could not give an educated guess when prompted.

### Subsets of education level correlated with percent increase in anemia knowledge score

**Figure 3:**
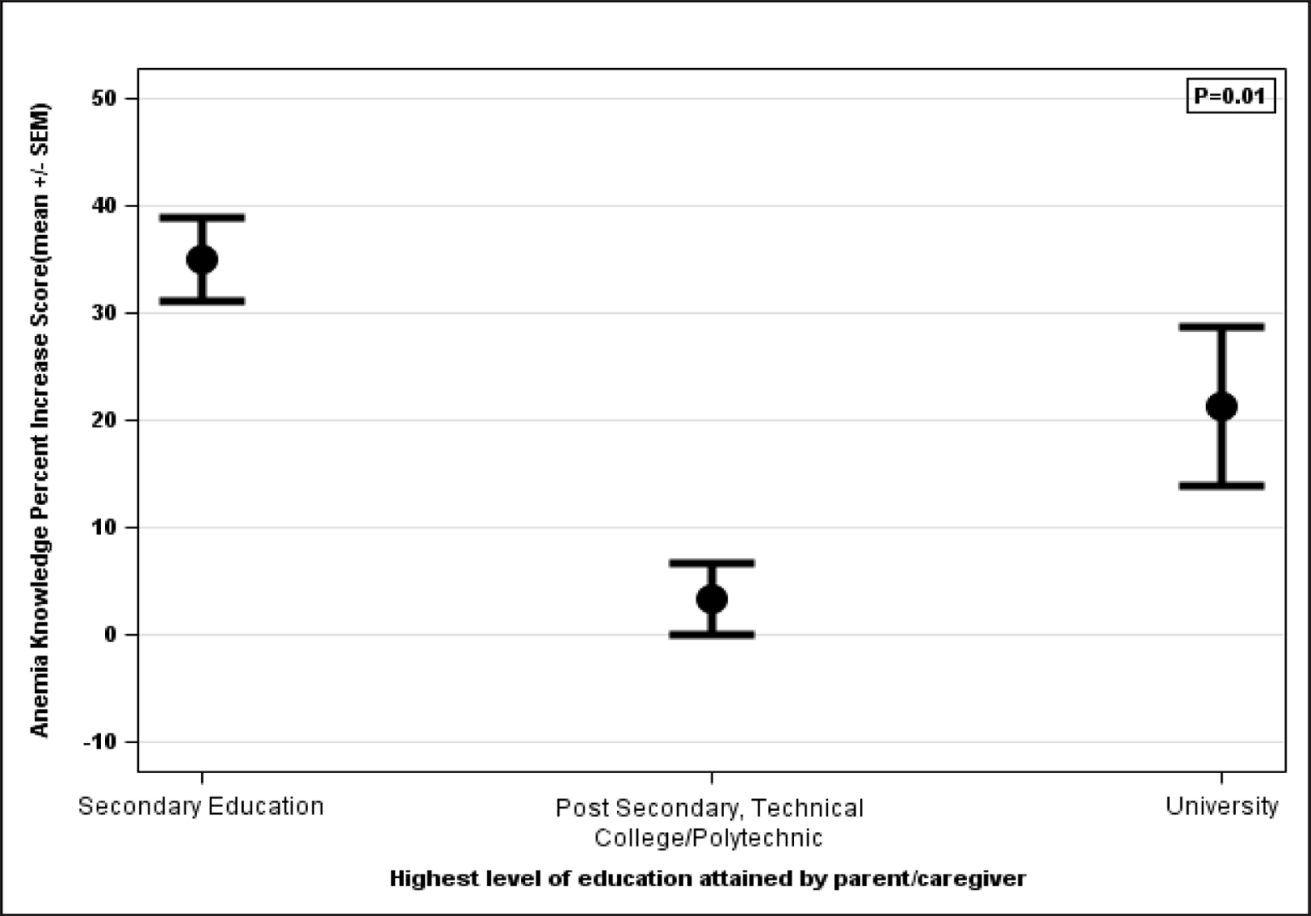
Comparison of percent increase in score based on education.

SES and highest level of education were both significantly associated with each other (P = 0.009); however, only subgroupings of education level correlated with survey results. The average percent increase in score between the pre- and post-education surveys for the anemia knowledge quiz was associated with the caregiver’s highest level of education received (Figure 3). The difference in percent increase in score was significant between a caregiver who had completed secondary education compared to postsecondary/technical college/polytechnic school (P = 0.002) but not compared to university (P = 0.14). However, there was no significant difference in baseline knowledge of anemia between education levels (P = 0.39).

### Capabilities scoring

**Figure 4:**
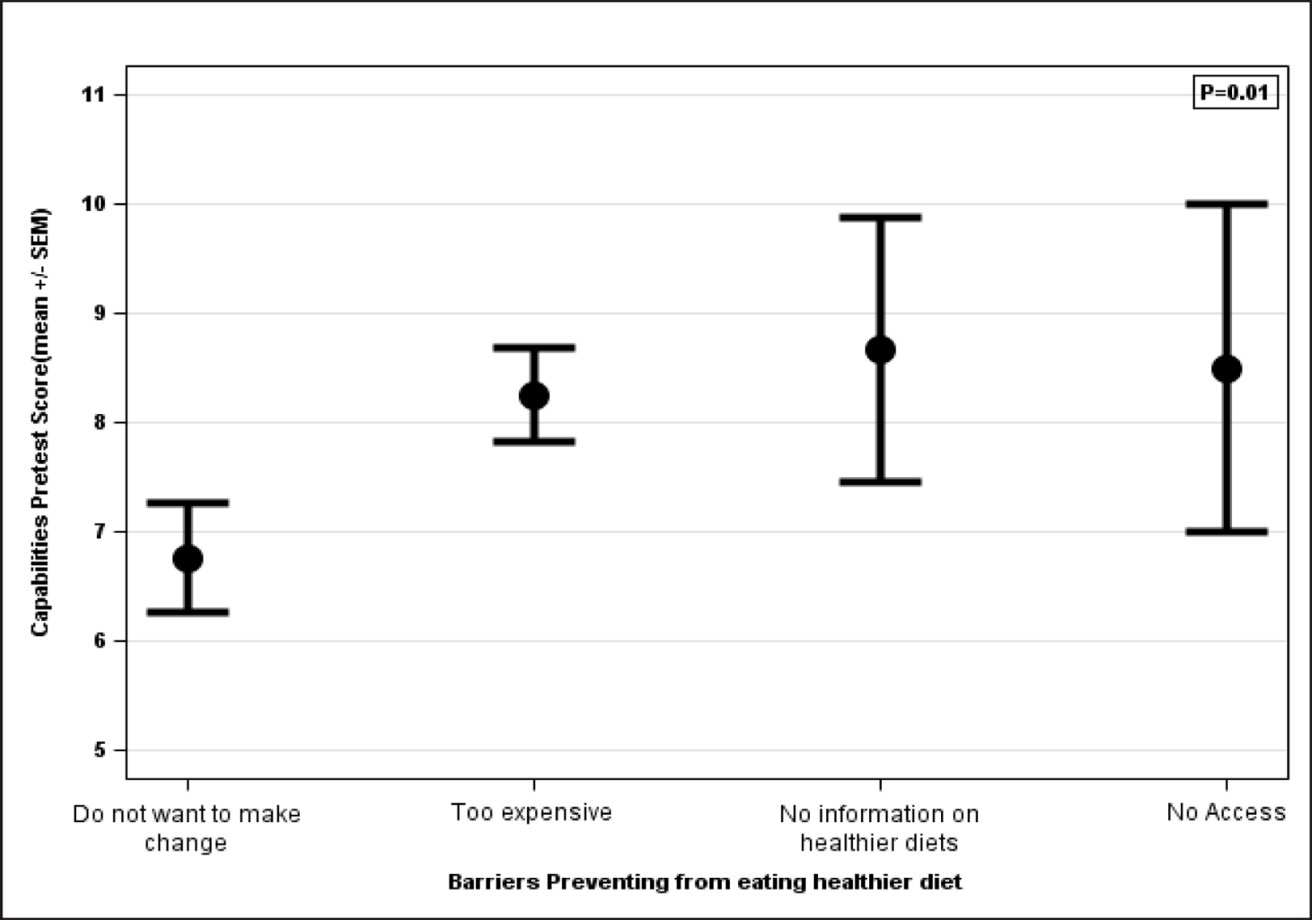
Association between pre-education capabilities scores and barriers to eating healthier diets.

**Figure 5:**
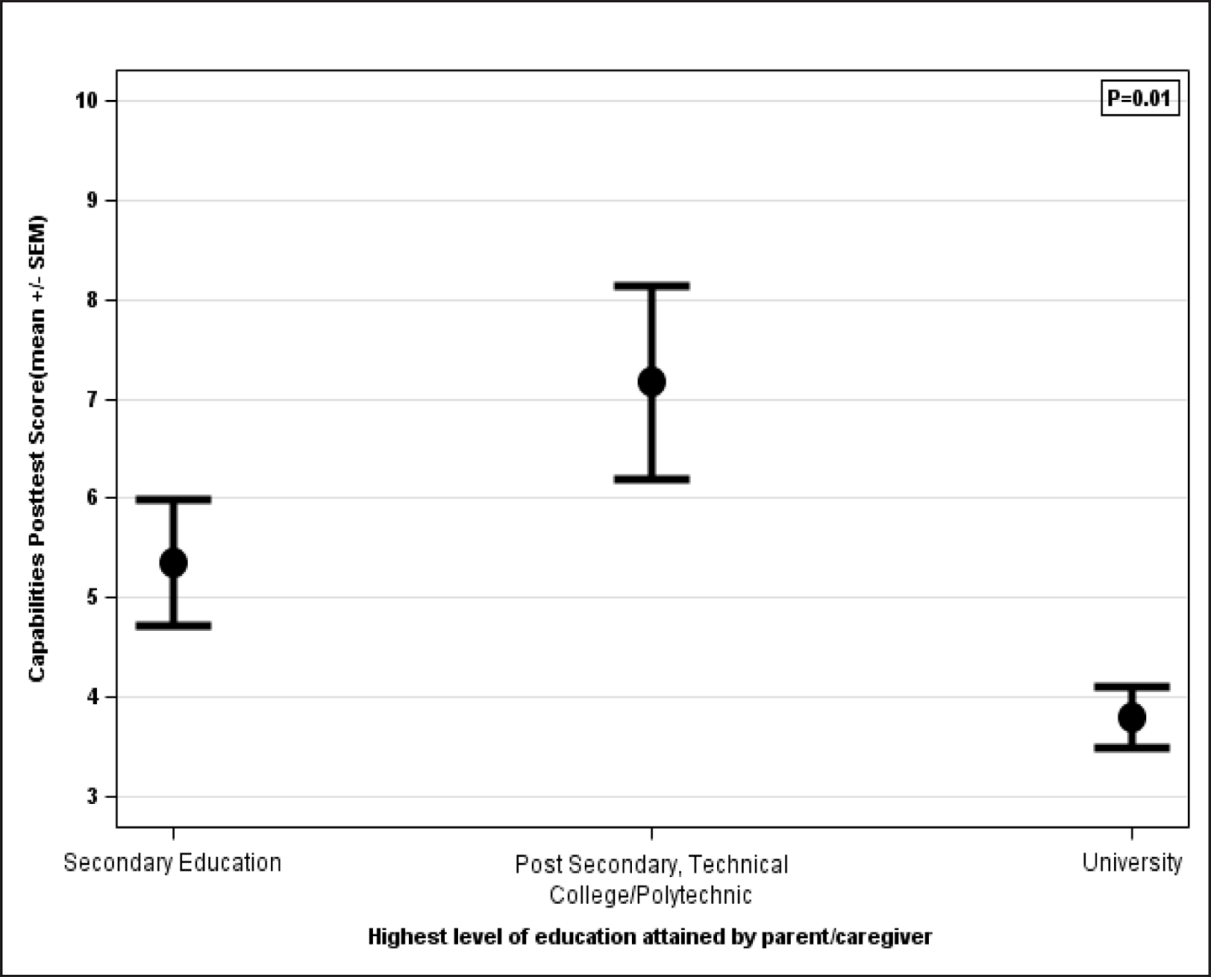
Association between post-education capability scores and caregiver’s highest education level.

The capability portion of the survey consisted of three questions assessing the level of independence or self-efficacy caregivers had in preventing IDA, improving or treating IDA, and adding iron-rich foods to their children’s diets (Appendix 1). Caregivers selected their level of independence on a 1–5 rating scale, with higher numbers indicating a decreased capability of accomplishing the task, and thus decreased self-efficacy. The average pre-education capability score before the education seminar was 8 out of 15 (53%), and the average post-education score was 5 out of 15 (33%), indicating an increase in self-efficacy of accomplishing the identified tasks. The pre-education capability scores were associated with the caregivers’ indicated barrier to eating a healthier diet (Figure 4). Caregivers who indicated higher self-efficacy and independence before the seminar were more likely to choose “don’t want to make a change” (Figure 5) as the main barrier for their family not eating a healthier diet.

While there was no association between each caregiver’s highest level of education received and their perceived capabilities in identifying and preventing IDA before the education seminar, one subgroup had significant statistical differences after the education seminar (Figure 5). The pre-education capability score for those who completed secondary education compared to university (P = 0.48) and those who completed postsecondary/technical college/polytechnic school compared to university (P = 0.35) were not statistically different. Similarly, after taking the educational seminar, the post-education capability score was only significantly different for those who completed postsecondary education compared to university (P = 0.005), but not for secondary school compared to either postsecondary education (P = 0.08) or university (P = 0.09).

## Discussion

While IDA is the most prevalent nutritional deficiency globally, there has been no firm consensus on the best manner by which to sustainably combat it [9,11,22,23,24,25,26]. However, due to the long-term cognitive, nutritional, growth, and immunological consequences of anemia in children, ascertaining methods to mitigate IDA in endemic low-resource regions is of especial importance [1,5,6,24].

Our study showed that across varying SES, deficiencies in knowledge of childhood anemia could be mitigated through the use of an education seminar. Our work provides preliminary evidence that empowering caregivers and providing them with basic knowledge about IDA and how to prevent it with local inexpensive and accessible iron-rich foods can foster a sustainable, long-term approach to combating pediatric IDA in this region.

Our study revealed poor knowledge of anemia regardless of SES or caregivers’ highest level of education. While SES was associated with timing of the most recent visit to a physician, there was no association found between the most recent medical visit and caregivers’ awareness of the child’s anemia diagnosis. Thus, our study highlights that even with access and means, barriers to health knowledge regarding pediatric anemia in the community persisted. This is in agreement with other studies in low- and middle-income countries (LMICs) that did not find a significant relationship between anemia severity and SES [5,19] and had low caregiver awareness of their child’s anemia [1,5,19]. These studies also assessed socioeconomic status by means of education level but did not have an interventional or education component for the parents [1,5]. This additional step is essential for both increasing the limited awareness caregivers have of their child’s anemia status and for providing a means to sustainably mitigate the prevalence of pediatric IDA.

In our study, families had moderate diet diversity, largely composed of 60% carbohydrates. There was no association found between diet diversity scores and the severity of a child’s anemia based on hemoglobin levels. This finding of diet homogeneity and lack of association between diet and anemia severity was comparable to a previous study in the same community [19]. Additionally, while SES was not associated with barriers to eating a healthier diet, it was associated with caregivers’ intended plan to incorporate iron-rich foods in their child’s diet. Our study also showed that 56% of caregivers reported either not wanting to make a change or not knowing what to buy as the largest barriers in eating a healthier diet, with 39% indicating price as the main factor. Collectively, this supports a lack of knowledge as a driving factor in poor diet diversity regardless of SES and identifies an area for targeted educational intervention that would increase the proportion of iron-rich foods in their family’s diet. While our study did not show an association with anemia severity and diet, the homogeneity of the population’s diet may be a factor in this. Further, the overall decreased diet diversity in the sampled community in an endemic region with known micronutritional deficiencies suggests a nutritional component to their children’s presenting with anemia. Other studies in the region and other LMICs have also noted a significant nutritional component to anemia severity in young children and highlighted the likelihood that increased growth requirements at the age coupled with low body iron stores and inadequate dietary patterns exacerbate this issue [1,5,7,27].

In our study, caregivers across education levels had comparable capability pretest scores and similarly poor knowledge of anemia and its risk factors. We found that after attending the education seminar, caregivers had statistically significant increases in anemia knowledge regarding what causes it and possible ways to prevent it. When further stratified, a caregiver’s level of education was associated with both the percent increase in anemia knowledge score and their capability post-test score. However, this was inconsistent between education levels for the two surveys. Self-efficacy of caregivers is an important factor to track, as research has shown that increasing self-efficacy of individuals provides greater chance of longitudinal and longer-lasting behavioral change [15,16,17,18]. The Sanitation Hygiene Infant Nutrition Efficacy (SHINE) trial is especially pertinent, as it identifies caregivers’ capabilities, also referred to as self-efficacy, as an effect modifier of an intervention’s impact [18]. However, this study only focuses on mothers’ self-capabilities and its interaction with utilizing resources to better their children’s health outcomes, while our study encompasses any type of caregiver [18].

Our results provide preliminary evidence that there are modifiable barriers to knowledge and access to optimal diet diversity, health literacy, and preventive health behaviors in resource-limited communities in southeast Nigeria. Additionally, it supports the feasibility of using a nutritional education program in this population across different socioeconomic and education levels to increase health literacy regarding IDA and ways to sustainably prevent it within a cultural context. While there was not a statistically significant difference in the increase in post-education capability scores after this initial seminar, the slight average increase in score suggests that with a more longitudinal approach, providers could continue to gain independence in identifying suitable iron-rich foods to supplement their families’ diets and reduce the risk of IDA in their children. This could also potentially mitigate the slight differences in educational subgroups for knowledge of anemia.

One limitation of the study was that the format used to test participants, a multiple-choice survey with options of “none of the above” or “all the above,” was somewhat difficult for participants, even though they were literate. It became apparent through conversation and continued administration that this form of testing is not common in the area, and for those who have not received higher levels of education, it may have proved especially cumbersome. Additionally, our study did not use definitive laboratory testing (i.e., peripheral blood smear) to test for anemia in children in the community. While this limitation was largely due to resource constraints, it means we cannot definitively conclude that IDA is the causative factor for anemia in this pediatric population. However, due to the endemic nature of multifactorial anemia in this region, an evidence-based assumption of iron deficiency being a large causative factor, based upon previous work in the community recording self-reported dietary nutritional constraints, is plausible. Further, iron supplementation in sub-Saharan Africa is recommended by the WHO for this population due to the endemic nature of IDA; thus, research ascertaining effective and sustainable models of ensuring this is still necessary [8,10,11,15,21].

## Conclusion

Our research shows that a community-centered, culturally sustainable nutrition intervention program would be accepted and could be longitudinally beneficial to mitigating pediatric IDA in this area.

The use of both a knowledge-based and capabilities assessment method is essential for evaluating behavioral change and to understand which factors affect how capable caregivers feel in enacting the tools and information provided to them. Our work is unique in combining both assessments within an education-based intervention program. In doing so, we hope to promote diet diversity and decrease the prevalence of pediatric IDA in this community in the future. A future study following both hemoglobin and iron-specific studies in conjunction with capability, knowledge, and diet diversity surveys over a defined follow-up time period would be important for assessing the impact of this intervention.

This community has a unique structure in that direct stakeholders in the community would be providing this health intervention, ensuring community trust, program adherence, and sustainability. Future studies evaluating the long-term impact of a targeted education program on improved diet diversity with iron-fortified foods and the anemia status of children in the community would be vital to combating pediatric IDA in this community and may improve overall health outcomes of children under age five.

Much of the hinderance to iron food fortification methods in LMICs is defining an adequate mode of delivery of knowledge of iron-fortified foods and access to them. This model would provide a sustainable method of decreasing pediatric IDA and increasing health literacy on the topic—an increasingly more favored approach than the traditional use of daily iron and/or vitamin supplements—thus empowering the community members to be active participants in theirs and their children’s health.

## Figures and Tables

**Appendix 1:**
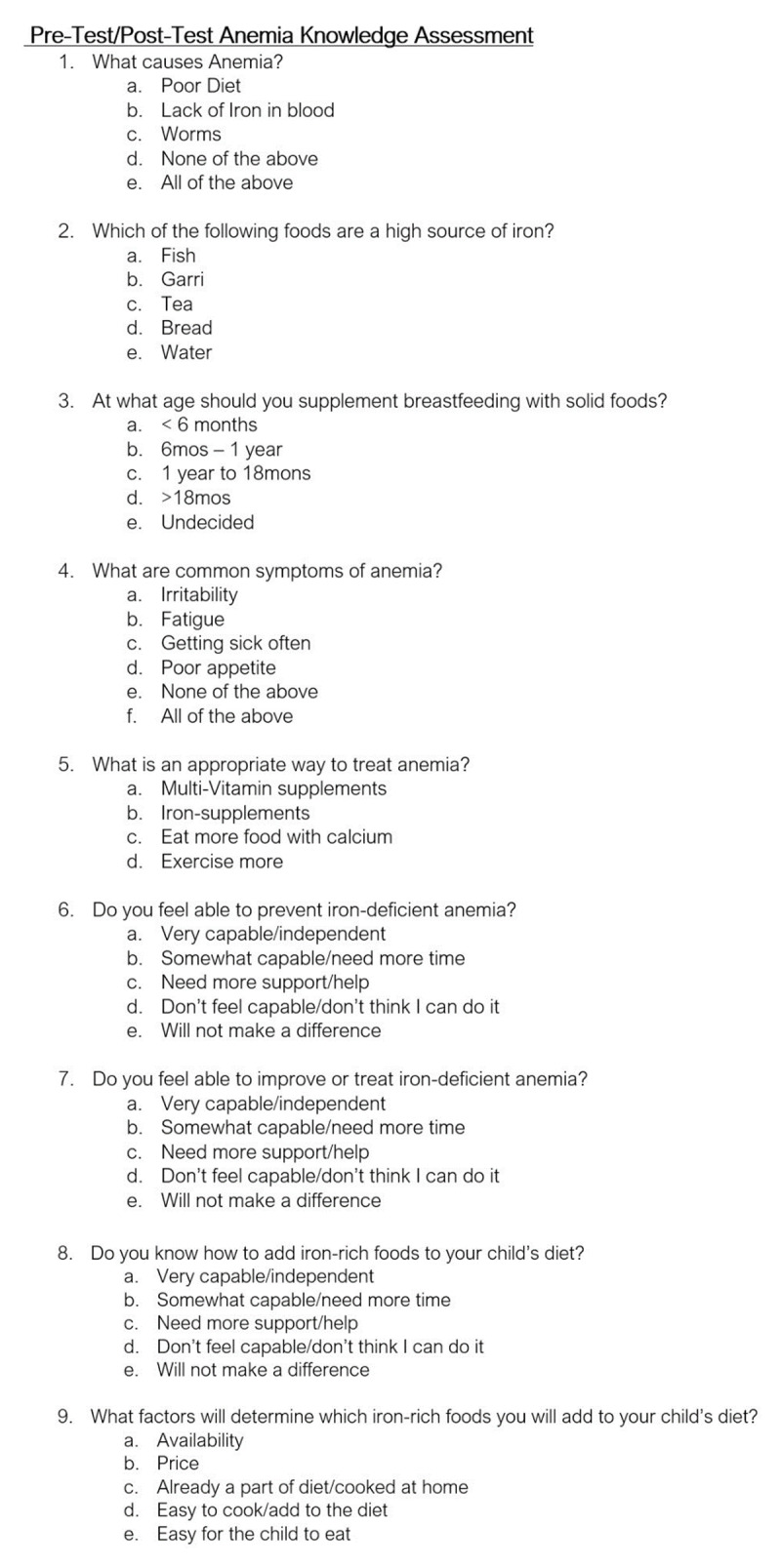
Pre- and post-education anemia knowledge survey.

**Appendix 2:**
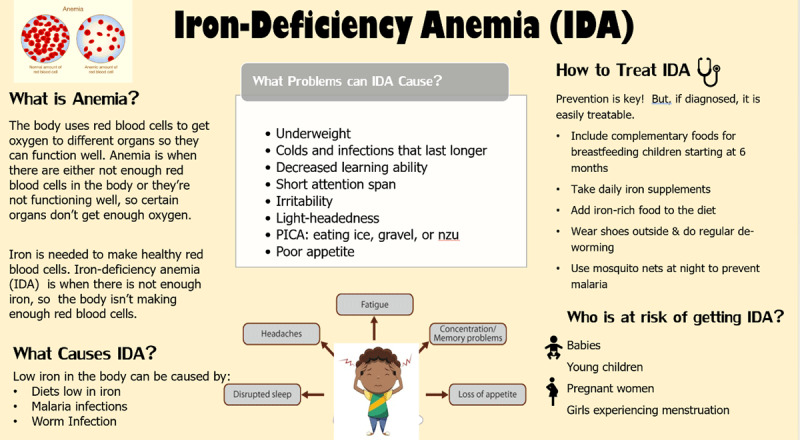
Health education seminar poster.

**Appendix 3:**
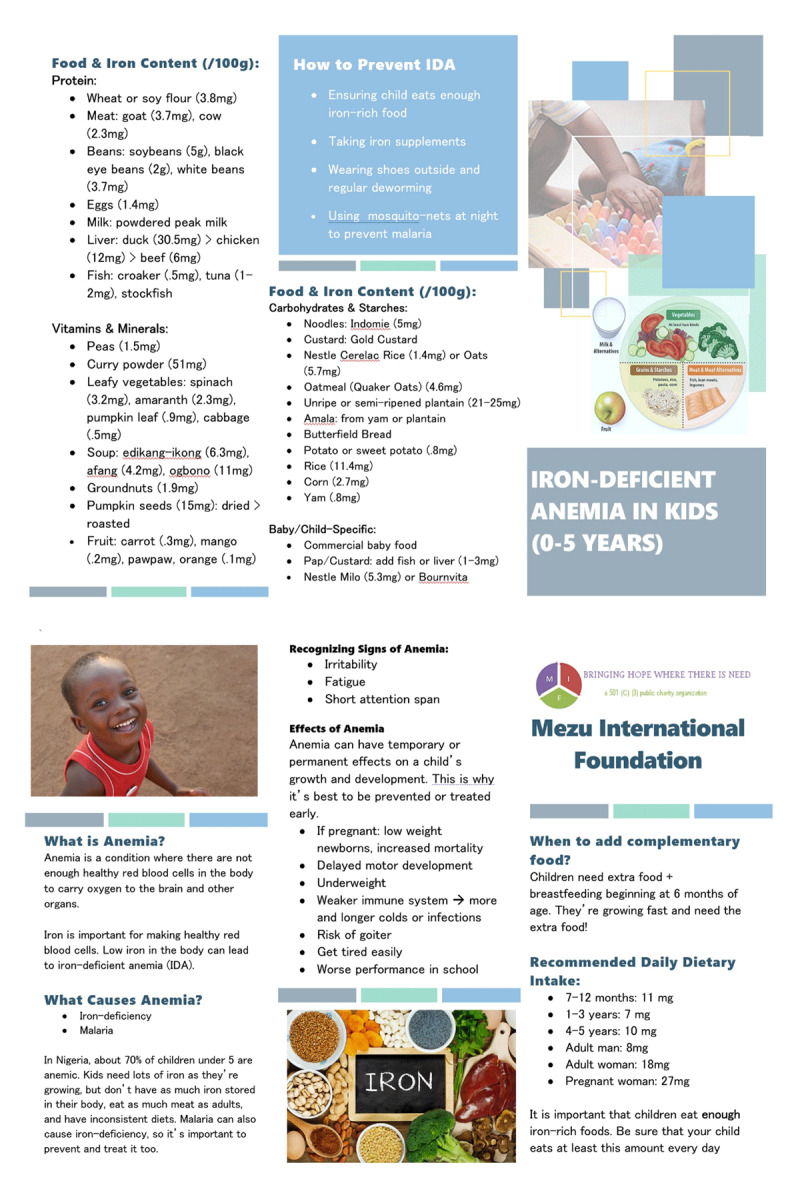
Pediatric iron-deficiency anemia caregiver brochure [27,28,29,30].
